# The high efficacy of luliconazole against environmental and otomycosis *Aspergillus flavus* strains

**Published:** 2020-04

**Authors:** Maryam Moslem, Ali Zarei Mahmoudabadi

**Affiliations:** 1 Infectious and Tropical Diseases Research Center, Health Research Institute, Ahvaz Jundishapur University of Medical Sciences, Ahvaz, Iran; 2 Department of Medical Mycology, School of Medicine, Ahvaz Jundishapur University of Medical Sciences, Ahvaz, Iran

**Keywords:** Antifungal susceptibility, Luliconazole, Amphotericin B, Voriconazole, Caspofungin, *Aspergillus flavus*

## Abstract

**Background and Objectives:**

Luliconazole is currently confirmed for the topical therapy of dermatophytosis. Moreover, it is found that luliconazole has *in vitro* activity against some molds and yeast species. The aim of the present study was to evaluate the efficacy of luliconazole in comparison to routine used antifungals on clinical and environmental isolates of *Aspergillus flavus*.

**Materials and Methods:**

Thirty eight isolates of *A. flavus* (18 environmental and 20 clinical isolates) were detected based on morphological and microscopic features and also PCR-sequencing of β-tubulin ribosomal DNA gene. All the isolates were tested against luliconazole, voriconazole, amphotericin B and caspofungin. Minimum inhibitory concentration (MIC), MIC_50_, MIC_90_ and MIC Geometric (GM) were calculated using CLSI M38-A2 protocol for both environmental and clinical isolates.

**Results:**

Luliconazole with extremely low MIC range, 0.00049–0.00781 μg/mL and MIC_GM_ 0.00288 μg/mL showed very strong activity against both clinical and environmental *A. flavus* isolates. Moreover, voriconazole inhibited 100% of isolates at defined epidemiological cutoff values (ECV ≤ 2 μg/ml). 50% and 27.8% of clinical and environmental isolates of *A. flavus*, were resistant to caspofungin, respectively. Whereas, all the isolates were found to be resistant to amphotericin B.

**Conclusion:**

The analysis of our data clearly indicated that luliconazole (with MIC_GM_ 0.00244 μg/ml for clinical and 0.00336 μg/ml for environmental isolates) had the highest *in vitro* activity against *A. flavus* strains.

## INTRODUCTION

Luliconazole, (−)-(E)-[(4R)- 4-(2, 4-dichlorophenyl)-1, 3-dithiolan-2-ylidene] (1H63imidazol-1-yl) acetonitrile, is a new synthetic imidazole antifungal. Luliconazole was firstly synthesized by Nihon Nohyaku Co Ltd in Japan in 2005 and similar to other common azoles, it is effective on ergosterol biosyn-thesis with fewer side effects and greater potency (1, 2). Luliconazole initially available as topical cream (LUZU) 1% for dermatophytosis and solution 10% for onychomycosis (1, 3, 4). A very low minimum inhibitory concentration (MIC) of luliconazole for dermatophytes, *Candida, Fusarium* and *Aspergillus* species has been reported (2, 5–7).

Amphotericin B has remained a Gold standard for the treatment of several invasive fungal infections for several decades (8, 9). Moreover, its fungicidal activity against the most of fungal isolates has been confirmed *in vitro* (10–12). Due to amphotericin B side effects and increased resistance to it, new antifungals for the treatment of disseminated mycosis were developed. During 2–3 past decades, new antifungals including, voriconazole, posaconazole and caspofungin were licensed for the treatment of invasive aspergillosis (7, 8). So that, voriconazole was presented as the first-line antifungal for invasive aspergillosis therapy (13, 14). Also, caspofungin is recommended for invasive aspergillosis in AIDs patients (5). The clinical resistance of *Aspergillus* species to echinocandins like caspofungin is very low (15).

*Aspergillus flavus* not only has the ability to causes primary infections in immunocompetent (16, 17), but also cause invasive infections in chemotherapy users, invasive therapy, immunocompromised patients, organ transplant and hematopoietic stem cell recipients (8, 14). In addition, *A. flavus* is one of the most important otomycosis agents (18). On the other hand, intrinsic and acquired azole - resistance have predominantly been reported for several *Aspergillus* species comprise *A. fumigatus, A. flavus, A. niger, A. terreus* and *A. lentulus in vitro* (2, 13, 19, 20). Moreover, resistant to amphotericin B in invasive aspergillosis has been reported for different *Aspergillus* species including *A. flavus* (21, 22). Due to limited information about the activity of luliconazole on *A. flavus* (7), in the present study we compared the efficacy of luliconazole vs. amphotericin B, voriconazole, and caspofungin against the clinical and environmental strains of *A. flavus*.

## MATERIALS AND METHODS

### Clinical and environmental isolates of *Aspergillus flavus*.

Twenty clinical isolates of *A. flavus* previously collected from otomycosis, were identified by morphological and microscopic characteristics and preserved in medical mycology laboratory affiliated to Ahvaz Jundishapur University of Medical Sciences. Furthermore, 16 strains of *A. flavus* collected from different areas of Ahvaz in autumn and winter 2018 using by Quick Tack air sampler (SKC 338.4530). In addition, two isolates of *A. flavus* were isolated from soil samples. All 38 *A. flavus* isolates were subcultured on Sabouraud dextrose agar (SDA) (Merck, Germany) supplemented with 0.05% chloramphenicol (Merck, Germany), and incubated at 29°C for 5 days. Then, strains were identified at the species level according to their macroscopic and microscopic morphological features. Color and texture of the *A. flavus* colonies were yellow green and cottony or powdery, respectively. Microscopic morphology include roughly and spiny conidiophores, loosely radiant phialides on most of vesicles and phialides to form uniseriate or biseriate were confirmed as *A. flavus* (23). This was approved by ethical committee of Ahvaz Jundishapur University of Medical Sciences (IR.AJUMS. REC.1398.263).

### Molecular identification and sequencing.

All isolates were subcultured on SDA and incubated at 29 °C for 3 days. Then, approximately, 300 mg of mycelia collected in microtubes containing 300 μl of lysis buffer and 50 mg glass bed (Sigma - Aldrich, USA) and were put at −20 °C for 24 h. Microtube contents homogenized by a SpeedMill PLUS Homogenizer (Analytikjena, Germany) were extracted using phenol-chloroform-isoamyl alcohol (Sigma - Aldrich, Germany) (24). PCR was performed, using primers Bt2a (5′-GGTAACCAAATCGGTGCTGCTTTC-3′) and Bt2b (5′-ACCCTCAGTGTAGTGACCCTTGGC-3′) for all isolates (25). The PCR products electrophoresed on agarose gel 1.2% and 500–600 bp bands were considered as *A. flavus*. Furthermore, 12 and 10 PCR products from environmental and clinical isolates were randomly selected and presented for nucleotide sequencing. Nucleotide sequence data aligned by Mega 6 Software were blasted using Gen-Bank database (100% similarity). Finally, the nucleotide sequence data were submitted to the GenBank database.

### Antifungal agents and antifungal assay.

A solution of luliconazole (APIChem Technology, China), voriconazole (Sigma-Aldrich, Ger many), caspofungin (Sigma-Aldrich, Germany), and amphotericin B (Sigma-Aldrich, Germany) were prepared in dimethyl sulfoxide (DMSO) (Merck, Germany) at 0.0001-0.125, 0.0625-8, 0.0312-4 and 0.5-64 μg/mL, respectively. *In vitro* antifungal susceptibility testing of 38 *A. flavus* isolates was performed using CLSI M38-A2 protocol (26). Briefly, a spore suspension of tested isolates was prepared in sterile 0.85% saline supplemented with 1% Tween 20 (Merck, Germany) and adjusted to 0.5 McFarland standard. Then, each microplate well was filled with 100 μL of each suspension and 100 μL of a serial dilution of each anti-fungals. Microtiter plates were incubated at 35 ºC for 24 h in humid incubator. Finally, MIC and minimum effective concentrations (MEC) were detected. The MIC_50_, MIC_90_ and MIC_Geometric(GM)_ were also calculated. The susceptibility (sensitive or resistant) was determined based on epidemiological cutoff values (ECVs) for amphotericin B (4 μg/ml), voriconazole (2 μg/ml) and caspofungin (0.5 μg/ml) (27).

### Statistical analysis.

The distribution of MIC between clinical and environmental *A. flavus* isolates was analyzed by χ ^2^ test and P values < 0.05 were considered statistically significant.

## RESULTS

In this study, according to microscopic and morphological features, 38 isolates of *A. flavus* were confirmed. Moreover, 22 of 38 isolates were randomly selected, sequenced and analysed. All sequenced data were deposited in Genbank (accession numbers; clinical isolates (10 isolates): LC440566 to LC440575; environmental isolates (12 isolates): LC457998 to LC458009). Table 1 presents the results of the *in vitro* susceptibility tests of four antifungal agents against 38 clinical and environmental isolates of *A. flavus*. As shown, luliconazole was exhibited a very low MIC against all tested *A. flavus* isolates, MIC = 0.00049–0.00781 μg/mL for clinically and MIC = 0.00195–0.00781 μg/mL for environmental isolates. Furthermore, as shown MIC_GM_ for clinical and environmental isolates was 0.00244 μg/mL and 0.00336 μg/mL, respectively.

**Table 1.** The antifungal susceptibility pattern of *Aspergillus flavus* isolates

***Aspergillus flavus***	**Antifungal**	**Minimum inhibitory concentration (μg/mL)**				**R (%)**	**%ECV^a^**

		**MIC**	**MIC_50/_**	**MIC_90/_**	**MIC_GM_**		
Clinical isolates (20)	LUL	0.00049–0.00781	0.00195	0.00391	0.00244	ND	ND
	AMB	16–64	32	64	44	20 (100)	0.0%
	CAS^b^	0.0625–4	0.25	2	0.24	10 (50)	50%
	VRC	0.0625–1	0.125	0.5	0.76	0 (0.0)	100%
Environmental isolates (18)	LUL	0.00195–0.00781	0.00391	0.00391	0.00336	ND	ND
	AMB	8–32	32	32	24.4	18 (100)	0.0%
	CAS^b^	0.0625–4	0.25	4	0.44	5 (27.8)	72.2%
	VRC	0.125–2	0.5	1	0.5	0 (0.0)	100%
All isolates (38)	LUL	0.00049–0.00781	0.00195	0.00391	0.00288	ND	ND
	AMB	8–64	32	64	33.8	38 (100)	0.0%
	CAS ^b^	0.0625–4	0.25	1	0.55	15 (39.5)	60.5%
	VRC	0.0625–2	0.25	0.5	0.34	0 (0.0)	100%

LUL, Luliconazole; AMB, Amphotericin B; CAS, Caspofungin; VRC, Voriconazole; GM, Geometric mean; R, Resistance; ND, not determined (no ECVs were available).

a %MICs less than or equal to than the epidemiologic cutoff values (ECVs) (ECV = 4 μg/ml for amphotericin B, 2 μg/ml for voriconazole, 0.5 μg/ml for caspofungin.

b Minimum effective concentration (MEC), MEC_50_, MEC_90_ and MEC_GM_ were calculated for *Aspergillus flavus*.

Although, the MIC range amphotericin B for environmental was lower (8–32 μg/mL) than clinical isolates (16–64 μg/mL), all strains (100%) were resistant to antifungal. Both clinical and environmental isolates of *A. flavus* inhibited at MEC range 0.0625–4 μg/mL of caspofungin, but resistant to caspofungin was more common among clinical isolates (50%) than environmental isolates (27.8%). The MIC range voriconazole for clinical and environmental isolates of *A. flavus* were 0.0625–1 and 0.125–2 μg/mL, respectively. As a results, 100% of isolates (environmental and clinical isolates) were sensitive to voriconazole. All isolates were found to be resistant to amphotericin B, whereas all clinical and environmental strains were sensitive to voriconazole.

Also, we did not found any statistically significant difference between clinical and environmental strains and resistant to caspofungin (P = 0.161713). In this study, only 10 (50%) and 5 (27.8%) clinical and environmental isolates of *A. flavus* were resistant to caspofungin, respectively. Moreover, it is found that there is a statistically significant difference between resistant to caspofungin and amphotericin B (P < 0.00001) and voriconazole and caspofungin (P = 0.000082). Resistance to two different classes of antifungals was only observed in amphotericin B and caspofungin (15 cases) (Table 2).

**Table 2.** The susceptibility pattern of *Aspergillus flavus* isolates

**Isolates**		**Number**	**LUL (μg/mL)**	**VOR**	**AMP**	**CAS**
Clinical isolates (20)	*A. flavus*	1	0.00781	S	R	R
	*A. flavus*	4	0.00391	S	R	R
	*A. flavus*	1	0.00391	S	R	S
	*A. flavus*	3	0.00195	S	R	R
	*A. flavus*	6	0.00195	S	R	S
	*A. flavus*	1	0.00098	S	R	R
	*A. flavus*	2	0.00098	S	R	S
	*A. flavus*	1	0.00049	S	R	R
	*A. flavus*	1	0.00049	S	R	S
Environmental isolates (18)	*A. flavus*	1	0.00781	S	R	S
	*A. flavus*	4	0.00391	S	R	R
	*A. flavus*	6	0.00391	S	R	S
	*A. flavus*	1	0.00195	S	R	R
	*A. flavus*	6	0.00195	S	R	S
Total		38	-	38S	38R	23S/15R

LUL, Luliconazole; VOR, Voriconazole; AMP, Amphotericin B; CAS, Caspofungin; R, Resistant; S, Sensitive

## DISCUSSION

*Aspergillus flavus* is a saprophytic filaments fungus with a high ability for causing different aspergillosis infections such as sinusitis, keratitis, invasive aspergillosis, aspergilloma, and otomycosis (19, 28). According to European Conference on Infections in Leukaemia (ECIL-6) guideline, voriconazole or isavuconazole are the first-line treatment of invasive aspergillosis in immunocompromised patients (29), whereas, in some cases the use of amphotericin B, is associated with treatment failure (21, 30).

Luliconazole was primarily presented for the treatment of onychomycosis, tinea pedis and tinea corporis by food and drug administration (FDA) (3, 4, 27), however it was recently found that it displays an excellent activity against several molds (*Aspergillus* and *Fusarium* species), yeasts (*Candida*) and dematiaceous fungi (2, 5–7, 31, 32). Luliconazole has a very low MIC against dermatophytes including *Trichophyton rubrum, T. mentagrophytes, T. tonsurans* and *Epidermophyton floccosum* (33). In the present study a novel antifungal drug, luliconazole, was used for the susceptibility evaluation of clinical and environmental *A. flavus* isolates *in vitro*.

Luliconazole was recently shown a potent *in vitro* activity against *Aspergillus* species including *A. fumigatus* (2), *A. terreus* (5), *A. flavus* (7) and *A. niger* complex (34) in comparison with other routine antifungal drugs. However, there is no data about the efficacy of luliconazole on *A. flavus* with otomycosis sources. In the present study, the strains of *A. flavus* isolated from otomycosis as well as environment strains were tested against luliconazole and the extremely low MICs (0.00049–0.00781 μg/mL) were obtained. Luliconazole with MIC_GM_ = 0.00288 μg/mL has shown the very high potent activity against clinical and environmental *A. flavus* strains. Similarly, in a study by Mahdavi-Omran et al. luliconazole showed the highest sensitivity to *A. flavus* strains in comparison with voriconazole, caspofungin and amphotericin B (7). Although, they found very low MIC_GM_ (0.008 μg/mL) for tested isolates, but our MIC_GM_ was highly low (MICGM 0.00288 μg/mL) for both sources, otomycosis and environmental isolates.

In our study all 38 tested isolates have shown that sensitive to voriconazole with the ECV ≤ 2 μg/ml. Many supportive studies have shown that *A. flavus* to be sensitive to voriconazole, like Denardi et al. and Mahdavi-Omran et al. which have reported MIC_GM_ values 0.871 and 0.27 μg/ml respectively (7, 11). Moreover, in a study by Borman et al. only 0.7% of *A. flavus* isolates were resistant to voriconazole (10) whereas all tested isolates by Varotto et al. were sensitive to voriconazole (35).

Echinocandin resistance is uncommon among *A. flavus* isolates. Diekema et al. Bedin Denardi et al. and Khodavaisy et al. have been reported excellent *in vitro* activity of caspofungin against clinical and environmental isolates of *A. flavus* (11, 12, 28). They found that all isolates had MEC_90_ lower than presented epidemiologic cutoff values. In our study the unexpected results, MEC ≥ 0.5 μg/ml for 10 clinical and 5 environmental isolates obtained for caspofungin. It seems that the source of isolates is effective on antifungal susceptibility. In this study, there was not any statistically significant difference between clinical and environmental strains and resistant to caspofungin (P = 0.161713).

Our results indicated that all isolates of *A. flavus* exhibited MICs ≥ 8 μg/ml against amphotericin B hence according to presented ECV all isolates were resistant to amphotericin B. Reichert Lima et al. have been reported that the 87% of *A. flavus* isolates from patients had MIC values ≥ 2 μg/ml and resistant to amphotericin B (8).

## CONCLUSION

In conclusion, the analysis of our data clearly indicated that luliconazole (with MIC_GM_ 0.00244 μg/ml for clinical and 0.00336 μg/ml for environmental isolates) had the highest *in vitro* activity against *A. flavus* strains. Furthermore, voriconazole and then caspofungin appeared to be good antifungal drugs against *A. flavus* with an acceptable rate of resistant isolates (15 isolates to caspofungin).
